# Impact of Silver Nanoparticles on Lemon Growth Performance: Insecticidal and Antifungal Activities of Essential Oils From Peels and Leaves

**DOI:** 10.3389/fpls.2022.898846

**Published:** 2022-05-23

**Authors:** Walid F. A. Mosa, Marwa I. Mackled, Nader R. Abdelsalam, Said I. Behiry, Abdulaziz A. Al-Askar, Adriana Basile, Ahmed Abdelkhalek, Mohsen M. Elsharkawy, Mohamed Z. M. Salem

**Affiliations:** ^1^Department of Plant Production (Horticulture-Pomology), Faculty of Agriculture, Alexandria University, Alexandria, Egypt; ^2^Department of Stored Product Pests, Plant Protection Institute, Agriculture Research Center, Alexandria, Egypt; ^3^Department of Agricultural Botany, Faculty of Agriculture, Alexandria University, Alexandria, Egypt; ^4^Department of Botany and Microbiology, College of Science, King Saud University, Riyadh, Saudi Arabia; ^5^Department of Biology, University of Naples Federico II, Naples, Italy; ^6^Department of Plant Protection and Biomolecular Diagnosis, Arid Lands Cultivation Research Institute (ALCRI), City of Scientific Research and Technological Applications, New Borg El Arab City, Egypt; ^7^Department of Agricultural Botany, Faculty of Agriculture, Kafrelsheikh University, Kafr El Sheikh, Egypt; ^8^Department of Forestry and Wood Technology, Faculty of Agriculture (El-Shatby), Alexandria University, Alexandria, Egypt

**Keywords:** lemon, AgNPs, yield and fruit quality, antifungal, *Fusarium solani*, insecticidal, *Sitophilus oryzae*, essential oils

## Abstract

Ten-year-old lemon (*Citrus limon* L. cv. Eureka) was used during the 2019 and 2020 seasons to investigate the effect of AgNPs at control, 5, 7.5, and 10 mg/L as a foliar application on vegetative growth, yield, and fruit quality. The selected trees were subjected to agricultural practices applied in the field during the study. The results indicated that the foliar application of AgNPs positively improved the shoot length, total chlorophyll, flower, and fruit set percentage, fruit yield, physical and chemical characteristics of fruits, and leaf mineral composition from macro and micronutrients compared to control in both seasons. The foliar application of AgNPs at 10 mg/L showed the highest mean values followed by 7.5 and 5 mg/L, respectively, for the previous characteristics. The treated leaves and fruit peels were hydrodistillated to extract the essential oils (EOs), and GC–MS analysis of leaf EOs. The analysis of leaves EOs showed the presence of neral, geranial, neryl acetate, and limonene as the main abundant bioactive compounds. While in peel the main compounds were neral, geranial, neryl acetate, D-limonene, geraniol acetate, linalool, and citronellal. Toxin effect of both EOs from leaves and peels were evaluated on the rice weevils (*Sitophilus oryzae*) and the results indicated a higher effect of lemon peel EOs than leaves based on mortality percentage and the values of LC_50_ and LC_95_ mg/L. *Melia azedarach* wood samples loaded with the produced lemon EOs were evaluated for their antifungal activity against the molecularly identified fungus, *Fusarium solani* (acc # OL410542). The reduction in mycelial growth was increased gradually with the applied treatments. The most potent activity was found in lemon leaf EOs, while peel EOs showed the lowest reduction values. The mycelial growth reduction percentages reached 72.96 and 52.59%, by 0.1% leaf and peel EOs, respectively, compared with control.

## Introduction

Lemon (*Citrus limon* L.) belongs to the Rutaceae family (Rahman et al., 2022). Worldwide, lemon is the third major cultivated citrus species after orange and mandarin trees (Pérez-Jiménez et al., 2022). The cultivated area in Egypt and the world is 14,947 and 1,226,617 hectares, producing 337,997 and 20,049,630 tons, respectively (FAO and UNICEF, 2019). As stated previously, the spray with a silver (Ag) minimized flower bud and flower abscission in orchids (Uthaichay et al., 2007). Ag ions could stop the action of ethylene by prohibiting its joining to the receptors in the cell of plants (Mishra et al., 2008). Nanoparticles (NPs) go inside the plant system and transfer *via* the vascular system (Corredor et al., 2009). AgNPs have a positive influence on improving the growth and development of plants (Shah and Belozerova, 2009; Jaskulski et al., 2022; Kannaujia et al., 2022; Mahajan et al., 2022). In combinations with carriers, NPs can be transferred inside the plant (Kurepa et al., 2010). NPs implementation supplies a new approach to increase plant productivity (Sharon et al., 2010; Dutta et al., 2022; Jaskulski et al., 2022). Many studies found that the linkage does the absorption of NPs in plant cells to the protein carrier through endocytosis and ions channels (Nair et al., 2010; Rico et al., 2011). Moreover, it was reported by Salama (2012) and Sharma et al. (2012) that AgNPs increased the length of shoots and roots, leaf surface area, leaf chlorophyll, contents of carbohydrate, and protein enzymes of antioxidants in *Brassica juncea* and common bean.

Essential oils (EOs) from peels and leaves of *C. limon* have identified several biological compounds and there are several findings displaying the strong insecticidal and repellent activity of Citrus peel EOs and their potential role as fumigants for stored-product insects (Saeidi et al., 2011; Ahmed et al., 2021; Akarca and Sevik, 2022; Ferrer et al., 2022). The most abundant compound was limonene from peels, followed by γ-terpinene, β-pinene, β-myrcene, and α-pinene (Michaelakis et al., 2009; Golmakani and Moayyedi, 2015). Limonene and neral with other compounds *trans*-verbenol, decanal, ethyl cinnamate, ethyl *p*-methoxycinnamate, *cis*-α-bergamotene, geraniol, trans-carveol, nonanal, linalool, and α-terpineol were identified in peel EOs (Paw et al., 2020). Compounds of limonene, β-pinene, neral, sabinene, geranial, geranyl acetate, neryl acetate, and geranyl acetate were identified in leaf EOs from *C. limon* (Vekiari et al., 2002; Hossain et al., 2021). Citrus essential oils and their compounds are informed to have multifarious activities, limonene is the main constituent compound (Mohamed and Mohamed, 2015; Khanikor et al., 2021). CEOs compounds having vital insecticidal potency tolerate all attributes to be utilized as green pesticides control of insect pests.

The rice weevil, *Sitophilus oryzae* L., of the Curculionidae family, wreaked havoc on several stored grains, including wheat, maize, and sorghum (Abdelsalam et al., 2019c; El-Naggar et al., 2020; Yang et al., 2020; Mosa et al., 2021; Abbas et al., 2022), which included reductions in nutritional quality, grain yield weight, and germination rates, besides their effect on human health and food safety because of allergen production. Several chemical insecticides are used as fumigants for stored pest control, such as methyl bromide, which was phased out and banned due to environmental issues and its hazardous effects on humans and the environment. In contrast, their thorough use has led to severe problems similar to insecticide resistance in several stored product insects like *Sitophilus* species (Fields and White, 2002; Ducom, 2012; Maletsema Alina, 2016; Pavlidi et al., 2018; Chelef et al., 2020). The other safe, effective, and eco-friendly methods are using alternative insecticides such as EOs and plant extracts to control insects’ stored products (Koul and Walia, 2009; Mamun and Ahmed, 2011; Mackled et al., 2019; Mahomed et al., 2019; Mosa et al., 2021; Ghareeb et al., 2022). The activity of insecticides on different citrus species has been demonstrated in numerous studies (El-Akhal et al., 2014; Abdelgaleil et al., 2015). *C. limon* has been established as an antifeedant, antimicrobial, antioxidant, insecticidal, and antifungal agent (Ali et al., 2017; Gucwa et al., 2018).

Several studies evoked the antifungal activity of EOs extracted from citrus trees (Mishra and Dubey, 1994; Sharma and Tripathi, 2006; Espina et al., 2011; Zohra et al., 2015). The studies reveal that EOs and their components have very significant antimicrobial potential.

This study was performed to investigate the impact of AgNPs on the vegetative growth, yield, and fruit quality of lemons. Besides, the effect of these treatments on the essential oil chemical composition of leaves and peels, in addition to their insecticidal and antifungal activities, was also investigated.

## Materials and Methods

### Experimental Design

This study was conducted during the 2019 and 2020 seasons. The lemon trees (*Citrus limon* L. Burm. F.) cv. *Eureka*, a 10-year-old, was previously planted 4 × 5 m apart in clay soil under a flood irrigation system in a private orchard located in the Rashid region, El-Beheira, Governorate, Egypt. The collection of *C. limon* specimens has been done with the permission of the private landowner. The physicochemical characteristics of the experimental soil are shown in Table 1. Twenty trees similar in vigor and size were randomly chosen as possible and arranged in a randomized complete block design (RCBD) (Sparks et al., 2020). The trees were subjected to agricultural practices in the field during the study. Before proceeding with the experiment, the physicochemical characteristics of the soil are determined.

**TABLE 1:** Physicochemical analysis of the experimental soil.

Parameters	0–40	40–70	Unit
Mechanical analysis			
Sand	87.52	78.8	%
Silt	2	2	%
Clay	10.48	19.2	%
Textural class	Sandy loam		
pH (1:2)	8.3	8.1	–
EC (1:1,water extract)	0.69	0.369	dS/m
O.M	0.14	0.5	%
Caco_3_	0.7	0.5	%
Soluble cations			
Ca^2+^	2	2	meq/L
Mg^2+^	2	3	meq/L
Na^+^	1.30	1.63	meq/L
K^+^	0.22	0.19	meq/L
Soluble anions			
HCO_3_^–^	3.3	2.2	meq/L
Cl^–^	1.23	1.6	meq/L
SO_4_^2–^	1.15	1.36	meq/L
Macronutrients			
Nitrogen (N)	8.5	9.4	mg/kg
Phosphorus (P)	10	21.5	mg/kg
Potassium (K)	275	750	mg/kg

*EC, electrical conductivity**;** O.M., organic matter.*

**FIGURE 1:**
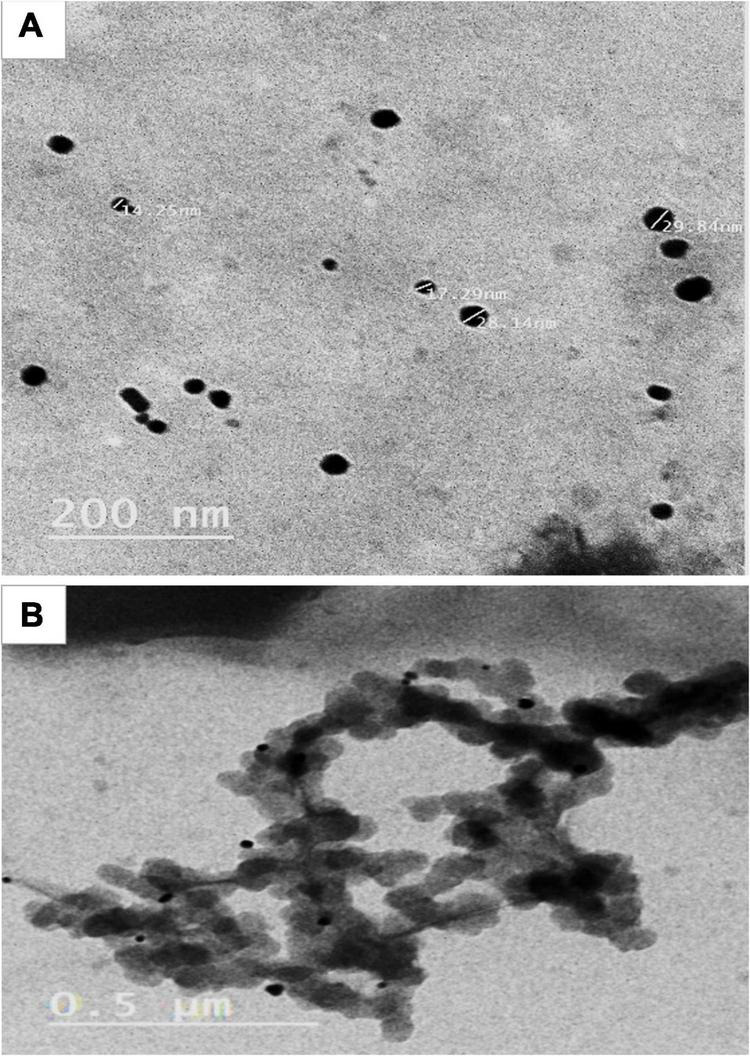
**(A,B)** TEM of AgNPs at low and high magnifications power in different scales.

To study the effects of AgNPs as a foliar application on lemon tree growth performance (vegetative growth, yield, and fruit quality), AgNPs were characterized for their particle shape by TEM (see Figure 1) and were prepared at the concentrations of (control, water only), 5, 7.5, and 10 mg/L. All the treatments were applied to the trees three times starting from mid-March with intervals of 1 month, for a total of 4 L for each tree/replicate.

### Vegetative Growth Parameters

Four shoots from each side, from each replicate/tree were selected and labeled. In contrast, the shoot length, and its diameter were measured in centimeters at the end of the growing season. Total chlorophyll in the fresh leaves was determined as SPAD units using the Minolta chlorophyll meter (SPAD, 502).

### Flower Number, Fruit Set Percentage, June Drop Percentage, and Fruit Yield

Four branches from each side were selected carefully (March 2019 and 2020). The flowers number on each branch was counted, and the fruit % was assessed according to the following formula:


$$\mathrm{Fruit}\mathrm{set}\left(\%\right)=\frac{\text{Number}\,\text{of}\,\mathrm{set}\,\mathrm{fruitlets}}{\text{Number}\,\text{of}\,\text{opened}\,\text{flowers}}\times 100$$

June drop percentage: In May 2019 and 2020, the dropped fruits were counted, and their percentage rate was estimated using the following formula:


$$\mathrm{June}\mathrm{drop}\left(\%\right)=\frac{\text{Number}\,\text{of}\,\mathrm{dropped}\,\mathrm{fruitlets}}{\text{Number}\,\text{of}\,\mathrm{set}\,\mathrm{fruitlets}}\times 100$$

Fruit yield: At the time of harvest in October, the yield of each treatment was estimated as a fruit weight (kg/tree and ton/hectare).

### Fruit Quality Measurements

In October, which is the harvest time, twenty fruits from each tree or replicate were chosen randomly and taken to the laboratory to estimate their physical and chemical fruit characteristics. Fruit weight (g) was calculated by recording the average weight of 20 fruits from each tree/replicate. Average fruit length (L) and fruit diameter (D) were measured using a hand caliper. Fruit volume (cm^3^) was calculated by dipping the fruit in water and weighing the removed water. Also, the weight of the peel (g) and flesh (g) was estimated. A Magness and Taylor pressure tester measured fruit firmness (Ib/inch^2^) with a 7/18-inch plunger.

The total soluble solids percent (T.S.S.%) was measured by using a hand refractometer (ATAGO Co., LTD., Tokyo, Japan) on the fresh-cut lemon fruit, and the result was expressed as a percentage (%). Total and reducing sugars were estimated calorimetrically using the Nelson arsenate–molybdate colorimetric method (Nielsen, 2010). Non-reducing sugars were measured by the difference between total sugars and reducing sugars. The percentage of titratable acidity in fruit juice was determined using an AOAC method (AOAC, 2005), where it was expressed as g citric acid/100 ml of fruit juice. The TSS/acid ratio was calculated by dividing the value of TSS over the value of titratable acidity. Ascorbic acid (vitamin C) content of the juice was estimated by titration with 2,6-dichloro phenol-indo-phenol (Nielsen, 2017) and calculated as mg/100 mg of juice.

### Chemical Composition of Leaves

For leaf chemical composition, samples of 30 leaves taken from the middle part of the shoots (Arrobas et al., 2018) were randomly selected from each replicate after the harvest time in November to determine their chemical composition of nitrogen (N), phosphorus (P), and potassium (K). The leaf samples were washed with tap water and then with distilled water and dried at 70°C until a constant weight was obtained. Finally, the dried leaf samples were ground, and acid digested using 12–15 ml of concentrated sulfuric acid (H_2_SO_4_), and 5 ml H_2_O_2_ for each gram until the digested solution became clear. The digested solution was used for the determination of nitrogen using the micro-Kjeldahl method (Wang et al., 2016), phosphorus by the vanadomolybdate method (Weiwei et al., 2017), and potassium using a flame photometer (Mutalik et al., 2011). Zinc, manganese, and iron were determined using an atomic absorption spectrophotometer (2–8,200 Series Polarized Zeeman, Hitachi, Tokyo, Japan) through specific lamps for specific nutrients as described previously (García et al., 2002; Hernández et al., 2010).

### Lemon Leaves and Peels Essential Oils Hydrodistillation and GC–MS Analysis

Leaves and peels from lemon trees were collected in November 2020. The raw materials were transferred into small pieces, then approximately 100 g from each were inserted into a flask (capacity, 2 L) containing 1,500 ml of distilled water (DW). The flask with its contents was heated under refluxing to hydrodistillate the material and extract the essential oil (EOs) using a Clevenger apparatus for 3 h (Okla et al., 2019; Elgat et al., 2020). The collected EOs were stored in brown glass bottles in a refrigerator at 4°C. The chemical constituents of the EOs from lemon peels and leaves were analyzed using a GC-TSQ Quantum mass spectrometer (Thermo Scientific, Austin, TX, United States) with a direct capillary column TG-5MS (30 m × 0.25 mm × 0.25 μm film thickness). The conditions for the separation and identification of the EOs can be found in the previous work (Abdelsalam et al., 2019c).

### Effect of EOs on *Sitophilus oryzae* Weevils by Contact and Residual Application Methods

*S. oryzae* weevils were reared under laboratory conditions (27 ± 1°C and 65 ± 5% RH) using sterilized wheat grains according to the methods (Mackled et al., 2019). Unsexed (male and female) adult weevils about 1–2 weeks old were used in this study. All experimental procedures were carried out under the same conditions as culture.

Contact and residual application methods evaluated the insecticidal activities of *C. limon* EOs (leaves and peels). The extracted EOs from the collected leaves and peels of lemon trees treated with AgNPs at different concentrations of control, 5, 7.5, and 10 mg/L were prepared at several doses (leaves: control, 20, 30, 40, 50, 60, 70, and 80 μl and peels: control, 5, 10, 15, 20, and 25 μl) were applied to filter paper (2.5 cm diameter). The treated filter paper was put into a Petri dish (Hyundai micro, Anseong, South Korea, 6.0 cm), and 20 adults of *S. oryzae* were released into the Petri dish, then covered by Parafilm. Control had a filter paper without any treatment. The plates were held under the same conditions as the control. All treatments were replicated three times (Broussalis et al., 1999; Peien et al., 2000; Wahba et al., 2018). Mortality percentages (M%) were determined after 48 h, and LC50 and LC95 values were calculated.

### Antifungal Activity of Wood-Treated With Essential Oils

The lemon root rot samples were collected from Rashid orchards, El-Beheira Governorate, Egypt. The isolation process revealed a fungus was initially classified according to morphologic characteristics and analyzed by DNA sequence data generated from the ITS regions of the rRNA genes (Al-Huqail et al., 2019).

The isolated fungus was used for the antifungal bioassay. Briefly, *Melia azedarach* wood samples were air-dried and prepared with an approximate dimension of 0.5 × 1 × 1 cm. The prepared wood samples were autoclaved for 20 min at 121°C, and then left to cool. Three wood samples were used for each treatment, as well as for Rizolex (1 g/L), the positive (10%, w/w tolclofos-methyl) and negative (10% DMSO) controls. The antifungal activity of the wood-treated in terms of the reduction rate percentage of fungal growth (RPFG) was measured following our previous works (Al-Huqail et al., 2019; Salem et al., 2019; Behiry et al., 2020; EL-Hefny et al., 2020) using the following formula; *RPFG*(%)[(*RC*-*RT*)/*RC*]100, where RC and RT represent the average diameters of the fungal growth of control and treatment, respectively.

### Statistical Analysis

The obtained data were subjected to one-way ANOVA according to Ott and Longnecker (2015), and the least significant difference (LSD) at 0.05% was utilized to compare the means of treatments. and measured with CoHort Software (Pacific Grove, CA, United States).

## Results

### Growth Parameters and Fruit Yield

Table 2 shows that the shoot length and diameter significantly increased by spraying AgNPs at 10 mg/L over the other applied treatments. Moreover, they were improved considerably by the foliar application of AgNPs at 5 or 7.5 mg/L compared with control in both seasons. Spraying AgNPs significantly raised the leaf’s total chlorophyll compared with control, and the highest increment was noticed with the foliar spraying of AgNPs at 10 mg/L over the usage of 7.5 or 5 mg/L. Flower number, fruit set percentage, and fruit yield per tree or hectare were significantly increased by the foliar application of AgNPs at 7.5 or 10 mg/L. They reduced the percentage of June drop compared with control or using AgNPs at 5 mg/L. Moreover, AgNPs at 10 mg/L treatment was more effective than 7.5 mg/L on the aforementioned parameters in the two experimental seasons.

**TABLE 2:** Influence of AgNPs spraying on shoot length, shoot diameter, leaf chlorophyll, flower number, fruit set %, June drop %, and yield of “Eureka” lemon during 2019 and 2020 seasons.

Treatment	Increase in shoot length (cm)	Increase in shoot diameter (mm)	Leaf chloro- phyll (SPAD)	Flower number	Fruit set %	June drop %	Yield (kg/ tree)	Yield (ton/ hectare)
**2019**								
Control (AgNPs 0 mg/L)	5.30*^d^ ± 0.45	0.11^c^ ± 0.01	55.14^d^ ± 3.04	814^c^ ± 8.68	28.61^c^ ± 1.99	24.15^a^ ± 2.15	84.4^d^ ± 3.21	21.1^d^ ± 0.80
AgNPs 5 mg/L	6.40^c^ ± 0.59	0.12^c^ ± 0.01	60.33^c^ ± 3.21	851^c^ ± 12.30	32.61*^b^* ± 0.66	22.08*^b^* ± 1.27	97.6^c^ ± 5.59	24.4^c^ ± 1.40
AgNPs 7.5 mg/L	7.64^b^ ± 0.72	0.15^b^ ± 0.01	65.36^b^ 2.91	908^b^ ± 25.53	37.50^a^ ± 2.56	18.03^c^ ± 1.10	108.6^b^ ± 5.46	27.15^b^ ± 1.36
AgNPs 10 mg/L	8.98^a^ ± 0.43	0.18^a^ ± 0.02	68.7^a^ ± 2.12	1,021^a^ ± 71.11	38.22^a^ ± 0.85	17.37^c^ ± 1.17	115.0^a^ ± 4.00	28.75^a^ ± 1.00
LSD_0.05_	0.75	0.01	3.08	44.02	2.60	1.99	4.68	1.17
**2020**								
Control (AgNPs 0 mg/L)	6.20^d^ ± 0.31	0.13^d^ ± 0.02	57.52^d^ ± 3.27	840.0^d^ ± 10	29.03^d^ ± 2.02	20.34^a^ ± 0.84	94.4^d^ ± 3.21	23.6^d^ ± 0.80
AgNPs 5 mg/L	7.26^c^ ± 0.29	0.17^c^ 0.02	64.15^c^ ± 2.86	883^c^ ± 28.26	32.99^c^ 0.58	18.33^b^ ± 0.85	107.6^c^ ± 5.59	26.9^c^ ± 1.40
AgNPs 7.5 mg/L	8.73^b^ ± 0.53	0.21^b^ ± 0.02	68.09^b^ ± 3.17	951^b^ ± 17.81	37.22^b^ 1.7	18.13^b^ ± 1.48	121.6^b^ ± 3.20	30.4^b^ ± 0.80
AgNPs 10 mg/L	9.85^a^ ± 0.45	0.24^a^ ± 0.02	72.60^a^ 2.26	1,021^a^ ± 53.20	43.8^a^ ± 2.07	17.08^b^ ± 0.63	128.8^a^ ± 5.97	32.2^a^ ± 1.49
LSD_0.05_	0.44	0.03	2.49	36.92	2.05	1.5	6.30	1.57

*Means not sharing the same letter(s) within each column, significantly different at 0.05 level of significance.

### Fruit Physical Characteristics

Table 3 shows that weight, size, length, diameter, and L/D ratio of fruit and flesh weight and fruit firmness as well as juice weight were statistically enhanced by spraying AgNPs as compared to control. It was also noticed that spraying AgNPs at 10 mg/L was more effective than the influence of 5 or 7.5 mg/L in the two seasons. The differences between AgNPs were obviously increased by increasing the used concentration, where 7.5 was better than 5 mg/L and, in the second season, more than the first one on the previously mentioned parameters.

**TABLE 3:** Influence of AgNPs spraying on “Eureka” lemon fruit weight, size, length and diameter, shape index, flesh weight, fruit firmness, and fruit juice weight during 2019 and 2020 seasons.

Treatment	Fruit weight (g)	Fruit size (cm^3^)	Fruit length (mm)	Fruit diameter (mm)	L/D Shape index	Flesh weight (g)	Fruit Firmness (Ib/inch^2^)	Juice weight (g)
**2019**								
Control (AgNPs 0 mg/L)	135.04*^c^ ± 3.25	153.33^c^ ± 4.62	65.45^c^ ± 2.39	55.74^d^ ± 1.65	1.17^c^ ± 0.01	80.13^c^ ± 7.82	10.73^d^ ± 0.25	39.02^d^ ± 1.06
AgNPs 5 mg/L	144.25^b^ ± 1.39	162.67^b^ ± 0.58	74.19^b^ 2.47	60.38^c^ 1.61	1.20^b^ ± 0.03	91.30^b^ ± 3.90	11.85^c^ ± 0.43	41.17^c^ ± 0.54
AgNPs 7.5 mg/L	151.48^b^ ± 4.71	169.67^b^ ± 3.51	75.59^b^ 3.10	62.86^b^ ± 1.08	1.23^a^ 0.01	100.15^a^ ± 4.54	12.93^b^ ± 0.51	42.93^b^ ± 0.53
AgNPs 10 mg/L	167.72^a^ ± 7.43	187.00^a^ 6.24	81.51^a^ ± 2.89	65.38^a^ ± 1.30	1.25^a^ ± 0.02	103.00^a^ ± 5.92	14.10^a^ ± 0.61	44.61^a^ ± 0.53
LSD_0.05_	8.16	7.26	1.55	0.89	0.02	4.98	0.59	0.94
**2020**								
Control (AgNPs 0 mg/L)	138.37^d^ ± 2.13	157.99^d^ ± 1.27	67.00^d^ ± 2.42	58.48^d^ ± 1.71	1.14^c^ ± 0.03	81.91^d^ ± 7.19	11.37^d^ ± 0.21	40.56^d^ ± 0.95
AgNPs 5 mg/L	146.89^c^ ± 1.38	165.48^c^ ± 0.78	75.18^c^ ± 1.69	62.07^c^ ± 2.48	1.21^b^ ± 0.05	93.02^c^ ± 2.17	12.57^c^ ± 0.29	42.83^c^ ± 0.97
AgNPs 7.5 mg/L	154.12^b^ ± 4.70	172.58^b^ ± 4.47	79.48^b^ ± 3.18	64.43^b^ ± 1.58	1.23^ab^ ± 0.03	104.47^b^ ± 3.04	14.17^b^ ± 0.49	45.32^b^ ± 0.79
AgNPs 10 mg/L	169.04^a^ ± 4.38	188.98^a^ ± 5.13	85.41^a^ ± 4.15	66.6^a^ ± 1.25	1.28^a^ ± 0.04	111.38^a^ ± 3.62	14.83^a^ ± 0.35	47.80^a^ ± 1.52
LSD_0.05_	5.8	5.95	3.24	1.45	0.06	5.82	0.52	1.83

*Means not sharing the same letter(s) within each column, significantly different at 0.05 level of significance.

### Fruit Chemical Characteristics

Table 4 shows that carotene content, vitamin C and TSS, as well as fruit acidity percentages, were significantly increased by spraying AgNPs at 5, 7.5, and 10 mg/L compared to control. The obtained results also showed that the impact of spraying AgNPs was increased in parallel with the increase in concentration, where AgNPs at 10 mg/L were higher than those at 7.5 or 5 mg/L. Total, reduced, and non-reduced sugars were minimized by the foliar spraying of AgNPs compared to control. Additionally, the concentration of 10 mg/L AgNPs was significantly higher than the foliar application of 5 or 7.5 mg/L in the two seasons.

**TABLE 4:** Influence of AgNPs spraying on “Eureka” lemon juice content from V.C., carotene and the percentages of TSS, acidity, total, reduced, and non-reduced sugars during 2019 and 2020 seasons.

Treatment	V.C. (mg/ 100 mg)	Carotene (mg/ 100 g)	TSS %	Acidity %	Total sugars %	Reduced sugars %	Non-reduced sugars %
**2019**							
Control (AgNPs 0 mg/L)	38.73*^b^ ± 1.27	2.34^d^ ± 0.06	8.22^c^ ± 0.08	5.77^c^ ± 0.08	1.85^a^ ± 0.05	1.19^a^ ± 0.01	0.65^ab^ ± 0.05
AgNPs 5 mg/L	40.73^ab^ ± 1.30	2.60^c^ ± 0.07	8.43^b^ ± 0.06	6.12^b^ ± 0.02	1.8^a^ ± 0.06	1.13^b^ ± 0.03	0.67^a^ ± 0.08
AgNPs 7.5 mg/L	42.33^a^ ± 0.91	2.76^b^ 0.03	8.53^ab^ 0.06	6.19^ab^ 0.02	1.72^b^ 0.03	1.12^b^ 0.02	0.59^bc^ ± 0.05
AgNPs 10 mg/L	42.57^a^ ± 1.97	2.95^a^ ± 0.12	8.7^a^ ± 0.1	6.33^a^ ± 0.15	1.62^c^ ± 0.05	1.07^b^ ± 0.02	0.54^c^ ± 0.03
LSD_0.05_	2.93	0.08	0.17	0.18	0.05	0.05	0.08
**2020**							
Control (AgNPs 0 mg/L)	38.11^c^ ± 1.55	2.50^d^ ± 0.05	8.53c ± 0.11	5.94^c^ ± 0.08	2.14^a^ ± 0.04	1.39^a^ ± 0.03	0.75^a^ ± 0.06
AgNPs 5 mg/L	41.71^b^ ± 2.16	2.65^c^ ± 0.05	8.80^b^ ± 0.2	6.39^b^ ± 0.12	2.09^a^ ± 0.01	1.34^ab^ ± 0.03	0.75^a^ ± 0.04
AgNPs 7.5 mg/L	44.62^ab^ ± 1.28	2.84^b^ ± 0.10	8.90^b^ ± 0.1	6.52^a^ ± 0.04	2.00^b^ ± 0.04	1.32^ab^ ± 0.02	0.67^ab^ ± 0.02
AgNPs 10 mg/L	46.47^a^ ± 1.15	3.05^a^ ± 0.13	9.07^a^ ± 0.06	6.59^a^ ± 0.12	1.91^c^ ± 0.03	1.30^b^ ± 0.04	0.61^b^ ± 0.03
LSD_0.05_	3.32	0.09	0.14	0.12	0.07	0.07	0.08

*Means not sharing the same letter(s) within each column, significantly different at 0.05 level of significance.

### Leaf Mineral Composition

Table 5 demonstrates that the leaf chemical composition of N, P, K, Fe, Zn, and Mn was raised considerably by spraying AgNPs at 5, 7.5, and 10 mg/L compared with control in the two experimental seasons. Besides, the leaf mineral content from these macro and micronutrients was obviously increased in parallel with increasing the used concentration of AgNPs, where 10 mg/L was better than 7.5 or 5 mg/L in both study seasons.

**TABLE 5:** Influence of AgNPs spraying on “Eureka” lemon leaf composition from N, P, K, Fe, Zn, and Mn during 2019 and 2020.

Treatment	N %	P %	K %	Fe mg/L	Zn mg/L	Mn mg/L
**2019**						
Control (AgNPs 0 mg/L)	1.41*^c^ ± 0.02	0.18^d^ ± 0.02	2.02^d^ ± 0.05	0.32^d^ ± 0.03	0.23^c^ ± 0.02	0.28^c^ ± 0.03
AgNPs 5 mg/L	1.49^b^ ± 0.02	0.22^c^ ± 0.01	2.37^c^ ± 0.03	0.36^c^ ± 0.02	0.38^b^ ± 0.03	0.47^b^ ± 0.02
AgNPs 7.5 mg/L	1.50^b^ ± 0.02	0.26^b^ ± 0.01	2.5^b^ ± 0.02	0.39^b^ ± 0.03	0.41^b^ ± 0.02	0.50^b^ ± 0.02
AgNPs 10 mg/L	1.53^a^ ± 0.02	0.30^a^ ± 0.02	2.69^a^ ± 0.03	0.42^a^ ± 0.02	0.45^a^ ± 0.02	0.65^a^ ± 0.02
LSD_0.05_	0.03	0.03	0.05	0.03	0.03	0.03
**2020**						
Control (AgNPs 0 mg/L)	1.48^d^ ± 0.02	0.23^d^ ± 0.01	2.15^d^ ± 0.04	0.36^d^ ± 0.02	0.3^d^ ± 0.03	0.33^d^ ± 0.03
AgNPs 5 mg/L	1.54^c^ ± 0.03	0.27^c^ ± 0.03	2.52^c^ ± 0.09	0.41^c^ ± 0.03	0.43^c^ ± 0.02	0.49^c^ ± 0.03
AgNPs 7.5 mg/L	1.58^b^ ± 0.02	0.31^b^ ± 0.02	2.60^b^ ± 0.05	0.47^b^ ± 0.03	0.48^b^ 0.04	0.54^b^ ± 0.03
AgNPs 10 mg/L	1.62^a^ ± 0.02	0.35^a^ ± 0.02	2.76^a^ ± 0.03	0.51^a^ ± 0.03	0.52^a^ ± 0.03	0.69^a^ ± 0.01
LSD_0.05_	0.04	0.03	0.07	0.04	0.04	0.03

*Means not sharing the same letter(s) within each column, significantly different at 0.05 level of significance.

### Insecticidal Activity of Essential Oils Against *Sitophilus oryzae*

**FIGURE 2:**
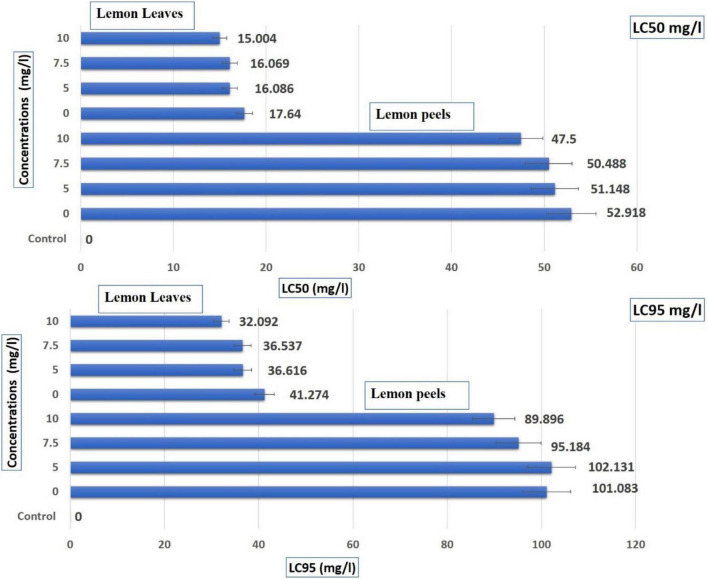
Toxic effect (contact and residual application methods) of lemon essential oils extracted from leaves and peels treated with AgNPs at control, 5, 7.5, and 10 mg/L, against *Sitophilus oryzae.*

Table 6 shows that the EOs from *C. limon* peels have a higher effect than leaves based on LC_50_ and LC_95_ (mg/L) values. The LC_50_ of leaves was 47.50 mg/L in the range of (40.23–56.09 mg/L) under 10 mg/L and 51.15 in the range (42.84–61.06 mg/L) under 5.0 mg/L and recorded 52.92 mg/L (EOs from untreated leaves) in the range of 44.70–62.64 mg/L (Table 6 and Figure 2). Additionally, the LC_95_ of leaves was 89.90 mg/L in the range (76.129–106.153 mg/L) under 10 mg/L and 102.131 mg/L in the range (85.536–121.945 mg/L) under 5.0 mg/L and recorded 101.083 (EOs from untreated leaves) in the range from 85.390 to 119.661 mg/L. In contrast, the LC_50_ values were lower with EOs from peels. They ranged from 15.00 mg/L in the range (11.86–18.98 mg/L) for EOs from trees treated with AgNPs 10–16.09 mg/L in the range (12.52–20.66 mg/L) for EOs from trees treated with AgNPs 5 mg/L, and 17.64 mg/L in the range (13.61–22.86 mg/L) for EOs from untreated peels. In point to LC95 of EOs from peels, 32.09 mg/L in the range of 25.36–40.61 mg/L as trees sprayed with AgNPs 10 mg/L, 36.616 in the range (28.503–47.038 mg/L) under AgNPs 5.0 mg/L, and recorded 41.274 mg/L (EOs from untreated leaves) in a range from 31.85 to 53.48 mg/L (Table 6 and Figure 2).

**TABLE 6:** Insecticidal activity of *C. limon* essential oil against *S. oryzae*.

Essential oils	Concentration of AgNPs (mg/L)	LC_50_ (mg/L)	Lower limit (mg/L)	Upper limit (mg/L)	LC_95_ mg/L (mg/L)	Lower limit (mg/L)	Upper limit (mg/L)	Slope ± SE
	Control	0	0	0	0	0	0	0
*C. limon* leaves	Control	52.918	44.702	62.643	101.083	85.390	119.661	4.565 ± 0.037
	5.0	51.148	42.837	61.072	102.131	85.536	121.945	4.282 ± 0.039
	7.5	50.488	42.783	59.580	95.184	80.659	112.326	4.663 ± 0.037
	10.0	47.500	40.226	56.090	89.896	76.129	106.153	4.644 ± 0.037
*C. limon* peels	Control	17.640	13.613	22.858	41.274	31.852	53.484	3.516 ± 0.057
	5.0	16.086	12.522	20.664	36.616	28.503	47.038	3.631 ± 0.055
	7.5	16.069	12.505	20.649	36.537	28.433	46.951	3.625 ± 0.056
	10.0	15.004	11.858	18.984	32.092	25.363	40.605	3.917 ± 0.052

Both EOs of *C. limon* from leaves and peels collected from untreated or treated trees with AgNPs showed a high mortality percentage against *S. oryzae*, as shown in Table 7. In leaves EO, the highest concentration (AgNPs 10 mg/L) showed an increase of10% in mortality percentage compared with control (81.67%) that registered (88.33%), and the other two concentrations (5 and 7.5 mg/L of AgNPs) showed 83.33 and 85.0%, respectively. With the increase in EO concentrations, there was an increase in the mortality percentage of *S. oryzae.* At the same time, the mortality percentage of *S. oryzae* under *C. limon* peels was lower than in leaves, ranging from 65.0% in control to 76.67% under the highest concentration of AgNPs (10 mg/L). Even though the mortality percentage was lower, the peel extract was more effective than the leaf extract based on the used concentration (Table 7).

**TABLE 7:** Mortality percentage of *C. limon* essential oil against *S. oryzae*.

Essential oils	Concentrations (mg/L)	Mortality percentage (M%) of essential oil from trees sprayed with			
		Untreated	AgNPs 5 mg/L	AgNPs 7.5 mg/L	AgNPs 10 mg/L
	0.0	0.00	0.00	0.00	0.00
*C. limon* Leaves	20.0	13.33	16.67	15.00	18.33
	30.0	20.00	25.00	21.67	28.33
	40.0	30.00	35.00	33.33	38.33
	50.0	45.00	46.67	48.33	53.33
	60.0	56.67	55.00	60.00	63.33
	70.0	70.00	71.67	73.33	76.67
	80.0	81.67	83.33	85.00	88.33
*C. limon* peels	0.0	0.00	0.00	0.00	0.00
	5.0	15.00	18.33	18.33	20.00
	10.0	30.00	38.33	36.67	43.33
	15.0	51.67	55.00	56.67	60.00
	20.0	58.33	66.67	63.33	70.00
	25.0	65.00	70.00	71.67	76.67

### Antifungal Activity of Lemon Leaves and Fruit Peels Essential Oils-Treated Wood Against the Isolated *Fusarium solani* Fungus

**FIGURE 3:**
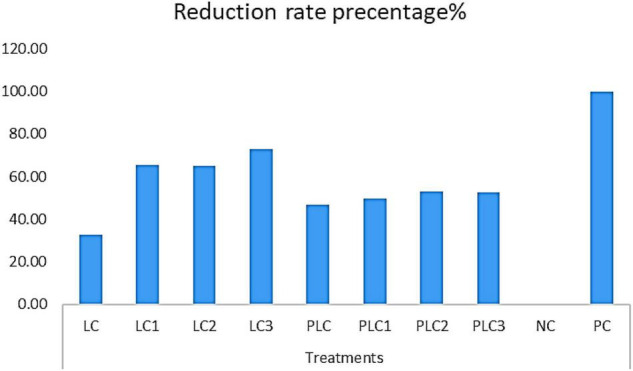
Antifungal activity of essential oils (0.1%) from leaves and peels of *Citrus limon* fruits collected from trees sprayed with Ag nanoparticles against the growth of *F. solani.* Treatments of leaves: LC, AgNPs 0 mg/L (control); LC1, AgNPs 5 mg/L; LC2, AgNPs 7.5 mg/L; LC3, AgNPs 10 mg/L. Treatments of peels: PLC, AgNPs 0 mg/L (control); PLC1, AgNPs 5 mg/L; PLC2, AgNPs 7.5 mg/L; PLC3, AgNPs 10 mg/L. NC, negative control (10% DMSO). PC, positive control (Rizolex 1 g/L).

**FIGURE 4:**
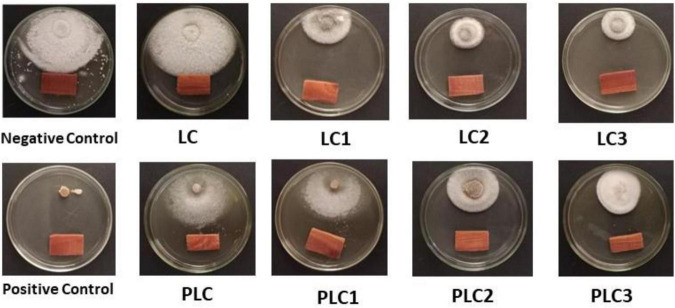
Antifungal activity of wood-treated with 0.1% leaves and peels essential oils from *Citrus limon* fruits sprayed with Ag nanoparticles with different doses. Treatments of leaves: LC, AgNPs 0 mg/L; LC1, AgNPs 5 mg/L; LC2, AgNPs 7.5 mg/L; LC3, AgNPs 10 mg/L. Treatments of peels: PLC, AgNPs 0 mg/L; PLC1, AgNPs 5 mg/L; PLC2, AgNPs 7.5 mg/L; PLC3, AgNPs 10 mg/L. Negative control (10% DMSO). Positive control (Rizolex 1 g/L).

The morphological and ITS region sequence revealed that the fungus was *Fusarium solani* (acc# OL410542). The antifungal activity of wood-treated EOs from leaves and peels of lemon trees as sprayed with different concentrations of AgNPs (5, 7.5, and 10 mg/L) in terms of reduction percentages of *F. solani* growth is shown in Figure 3. Figure 4 shows the visual observation of the antifungal activity of wood-treated with leaves and peels’ EOs as affected by the spraying of AgNPs. Overall, the results indicate that all EOs at a concentration of 0.1% from leaves reduced the linear growth of *F. solani*, with inhibition 32.59 ± 1.05%, 65.56 ± 0.91%, 65.19 ± 1.05%, and 72.96 ± 0.52% compared to the control. At the same time, peel treatments reduced the fungus growth by 46.67 ± 0.00%, 49.63 ± 0.52%, 52.96 ± 0.52%, 52.59 ± 0.52%, respectively, compared to the positive control used (Rizolex, 1 g/L) with an inhibition value of 100%.

### Chemical Composition of Essential Oils From Peels and Leaves

Table 8 presents the chemical compounds of EOs identified in *C. limon* leaves. The most abundant compounds were neral, geranial, neryl acetate, limonene, *cis-α*-bergamotene, and β-bisabolene. The changes in percentages of abundant compounds in the EOs from peels as affected by the application of water (control) and AgNPs at 5, 7.5 and 10 mg/L concentrations were 22.01, 16.94, 20, and 21.62%; 29.35, 19.2, 20.74, and 25.01%; 4.02, 4.12, 5.07, and 3.90, 19.97, 17.98, 16.39, and 18.79%; 5.25, 6.46, 6.28, and 4.79%; 2.8, 3.8, 4.4, and 3.31%; 2.91, 3.44, 3.85, and 3.10%; 9.33, 15.94, 11.5, and 8.63%, for neral, geranial, neryl acetate, limonene, bergamotene, geraniol acetate, β-caryophyllene, and β-bisabolene, respectively.

**TABLE 8:** Chemical composition of ECo from peels of *Citrus limon*.

Compound	Peel essential oil from trees treated with			
	Untreated	AgNPs 5 mg/L	AgNPs 7.5 mg/L	AgNPs 10 mg/L
*Cis*-Sabinene hydrate	ND	0.24 (863)	ND	ND
Isopulegol		0.71 (890)	0.65 (879)	0.66 (844)
D-Limonene	19.97 (912)	17.98 (874)	16.39 (864)	18.79 (878)
Boldione		0.21 (988)	ND	ND
(Z)-Citral (Neral)	22.01 (919)	16.94 (922)	20.00 (893)	21.62 (906)
(E)-citral (Geranial)	29.35 (931)	19.20 (934)	20.74 (928)	25.01 (931)
Citral	0.43 (858)	0.44 (935)	0.56 (937)	0.65 (846)
1,3,3-Trimethyl-2-vinyl-1-cyclohexene	ND	ND	1.43 (826)	ND
Neryl acetate	4.02 (912)	4.12 (929)	5.07 (926)	3.90 (929)
Geraniol acetate	2.80 (938)	3.80 (930)	4.40 (930)	3.31 (935)
β-Caryophyllene	2.91 (945)	3.44 (940)	3.85 (929)	3.10 (940)
*Cis*-α-Bergamotene	5.25 (959)	6.46 (953)	6.28 (957)	4.79 (958)
*Trans*-β-Farnesene	ND	1.14 (898)	1.32 (892)	1.01 (897)
Valencene	1.31 (919)	ND	1.03 (916)	2.19 (932)
Eremophilene	ND	0.90 (898)	ND	ND
β-bisabolene	9.33 (939)	15.94 (915)	11.50 (913)	8.63 (944)
Nerolidol	ND	ND	0.69 (894)	0.75 (894)
Viridiflorol	1.15 (830)	0.83 (785)	1.00 (829)	ND
Globulol	ND	ND	ND	1.05 (839)
*Trans*-Sesquisabinene hydrate	1.01 (813)	0.76 (813)	0.81 (815)	0.64 (797)
α-Bisabolol	0.90 (936)	0.76 (930)	0.74 (927)	0.61 (925)
Dimethyl myristamine	ND	3.06 (933)	1.31 (908)	ND
α-Bisabolol oxide A	ND	0.45 (856)	ND	ND
Oleic acid, methyl ester	ND	0.38 (881)	0.73 (807)	1.06 (827)
Linoleoyl chloride	ND	0.62 (831)	0.58 (800)	0.93 (842)

*ND, not detected.*

Table 9 shows the chemical compounds identified in the EOs from lemon leaves as affected by the foliar application of AgNPs. The changes in the abundant compounds neral, geranial, neryl acetate, D-limonene, geraniol acetate, linalool, citronellal, and caryophyllene were 15.58, 12.51, 18.64, and 16.24%; 15.44, 12.9, 20.41, and 17.15%; 17.61, 10.99, 15.70, and 17.62%; 12.71, 15.09, 12.38, and 13.95%; 9.06, 6.01, 9.05, and 9.45%; 3.73, 2.89, 3.81, and 4.10%; 2.57, 1.39, 1.68, and 2.05%; 3.58, 2.74, 3.26, and 2.94% as affected by foliar application of water (control) and AgNPs at 5, 7.5, and 10 mg/L concentration, respectively. Boldione compounds were detected in EO from control plants (5.32%) and peels from treated trees with AgNPs at 7.5 mg/L (5.97%). While, α-terpineol was detected in EO from peels collected from treated trees with AgNPs at 5 mg/L (3.14%) and AgNPs at 10 mg/L (4.72%).

**TABLE 9:** Chemical compositions of essential oils from leaves of *Citrus limon*.

Compound	Leaf essential oil from trees treated with			
	Untreated	AgNPs 5 mg/L	AgNPs 7.5 mg/L	AgNPs 10 mg/L
α-Pinene	ND	1.78 (943)	ND	ND
β-Pinene	0.42 (936)	9.35 (938)	0.08 (935)	0.18 (944)
Myrcene	ND	0.87 (924)	ND	ND
Linalyl acetate	ND	0.46 (911)	ND	ND
D-Limonene	12.71 (912)	15.09 (905)	12.38 (906)	13.95 (901)
(*E*)-β-Ocimene	ND	2.47 (936)	ND	ND
γ-Terpinene	ND	0.69 (945)	ND	ND
4-Thujanol	ND	0.47 (952)	ND	ND
p-Mentha-1,4(8)-diene	ND	0.29 (943)	ND	ND
Linalool	3.73 (902)	2.89 (913)	3.81 (912)	4.10 (892)
*Cis*-Limonene oxide	ND	0.40 (939)	ND	ND
Exo-Isocitral	ND	0.29 (919)	ND	ND
Citronellal	3.58 (923)	2.74 (923)	3.26 (925)	2.94 (921)
Isoneral	0.27 (821)	1.63 (897)	0.12 (928)	0.30 (919)
Boldione	5.32 (983)	ND	5.97 (974)	ND
Nerol	0.33 (922)	0.19 (919)	ND	0.12 (918)
Terpinen-4-ol	ND	0.29 (911)	ND	ND
Isogeranial	ND	2.57 (866)	0.23 (921)	ND
α-Terpineol	ND	3.14 (936)	ND	4.72 (807)
Decanal	ND	0.44 (857)	ND	ND
(Z)-Citral (neral)	15.58 (937)	12.51 (935)	18.64 (929)	16.24 (934)
Citrol (Geraniol)	ND	0.24 (776)	ND	ND
(E)-citral (Geranial)	15.44 (930)	12.9 (933)	20.41 (923)	17.15 (919)
Epoxy-linalooloxide	ND	0.17 (785)	0.08 (787)	0.07 (838)
Methylnaphthalene	0.30 (927)	0.14 (916)	0.45 (965)	0.22 (928)
Undecanal	0.78 (965)	0.49 (962)	0.46 (959)	0.64 (962)
2,3-Expoxycitral	ND	ND	0.59 (759)	0.30 (910)
γ-Dodecalactone	ND	0.90 (871)	ND	0.49 (867)
Citronellyl acetate	ND	1.85 (929)	0.44 (907)	0.54 (909)
Neryl acetate	17.61 (923)	10.99 (929)	15.70 (937)	17.62 (927)
Geraniol acetate	9.06 (941)	6.01 (937)	9.05 (930)	9.45 (936)
Caryophyllene	2.57 (940)	1.39 (957)	1.68 (936)	2.05 (939)
*Cis*-α-Bergamotene	0.37 (955)	0.22 (955)	0.29 (952)	0.26 (948)
Humulene	0.71 (940)	0.36 (863)	ND	0.25 (943)
(2,2,6-Trimethylbicyclo (4.1.0) hept-1-yl) methanol	0.19 (761)	0.28 (731)	ND	ND
Geranyl propionate	0.25 (905)	0.23 (901)	0.39 (876)	0.27 (893)
2,3-Epoxy-geranyl acetate	ND	ND	0.09 (893)	
(2Z)-3,7-Dimethyl-2-octenyl 2-methylpropanoate	ND	ND	0.68 (801)	0.20 (752)
Acenaphthene	0.17 (953)	ND	0.24 (955)	0.11 (941)
γ-Elemene	0.79 (896)	0.26 (893)	ND	0.50 (895)
α-Farnesene	2.46 (950)	0.87 (936)	0.69 (894)	1.00 (933)
Cadina-1(10),4-diene	0.28 (936)	0.17 (950)	0.10 (941)	0.18 (939)
Nerolidol	0.18 (946)	0.11 (937)	0.09 (940)	0.13 (951)
Spathulenol	1.82 (819)	0.86 (917)	0.78 (935)	1.82 (918)
Caryophyllene oxide	0.98 (895)	0.60 (920)	1 (925)	0.79 (904)
Ledene oxide-(II)	0.09 (814)	ND	ND	ND
Alloaromadendrene oxide-(1)	0.28 (840)	0.16 (836)	ND	0.22 (828)
Caryophylladienol II	0.29 (909)	0.13 (908)	0.12 (899)	0.18 (912)
.tau.-Muurolol	0.27 (930)	0.14 (937)	0.15 (838)	0.21 (925)
α-Cadinol	0.55 (897)	0.35 (909)	0.27 (892)	0.46 (906)
*Trans*-Sesquisabinene hydrate	0.38 (833)	0.23 (834)	0.20 (825)	0.10 (849)
α-Bisabolol	0.29 (954)	ND	0.16 (955)	0.23 (950)
Ledol	0.38 (812)	ND	ND	0.30 (846)
Phytol	0.48 (927)	0.36 (939)	0.36 (934)	0.58 (919)
Heneicosane	ND	ND	ND	0.15 (906)
Heptacosane	ND	ND	ND	0.12 (868)
Linoleic acid	ND	0.15 (880)	0.10 (815)	0.29 (853)
Oleic acid	ND	0.16 (882)	0.17 (856)	ND

*ND, not detected.*

## Discussion

The obtained results proved that AgNPs had a positive influence on improving the vegetative growth, yield, and fruit quality of lemon (*C. limon* L.). AgNPs are promising materials that have a good influence on the development of crops, particularly by improving the flowering rate, the proportion between root and shoot systems, the growth of seeds, and the formation and rigidity of roots (Ma et al., 2011; Fouda et al., 2020a; Dutta et al., 2022; Ghareeb et al., 2022; Mahajan et al., 2022). The application of NPs in the agricultural sector improved the resistance to diseases, increased the efficiency of plant elements uptake, pests’ control, raised the ecological pressures tolerance, and provided a processing and storage coefficient system to increase crop productivity (Mousavi and Rezaei, 2011). Several studies noticed that using AgNPs enhanced the size of root and shoot systems in plants, length and width of leaves, chlorophyll content, carbohydrates, proteins, and enzymes of antioxidants of *Brassica juncea*, bean, and wheat (Salama, 2012; Sharma et al., 2012; Fouda et al., 2020b). Jhanzab et al. (2015) and Abdelsalam et al. (2019a,b) stated that NPs greatly enhanced the uptake and transferring of NPK, which are the essential elements acquired for plant growth in many crops. Nano fertilizers can increase the contents of cellular chlorophyll, thereby increasing the rates of photosynthesis (Singh and Kumar, 2017). Still, on the opposite side, they minimize the undesirable impacts of biotic and abiotic stresses on seed germination (Tantawy et al., 2015; Giorgetti et al., 2019). Also, Singh et al. (2017) stated that using nanoparticles raised crop nutrient usage, minimized ecological pollution, and improved plant growth by introducing plant resistance and nutrient absorption. The application of *Lupinus termis* seedlings with silver nanoparticles at 100 μg/ml increased the growth parameters, but using 300 or 500 μg/ml negatively impacted the same parameters (Al-Huqail et al., 2018). Our study results are similar to the previous findings of Mosa et al. (2021), they found that the AgNPs foliar application at 10, 12.5, and 15 mg/L on peach trees can enhance the vegetative growth, the percentages of formed flowers, fruit set, and yield, as well as fruit physiochemical characteristics.

Peel EOs of *C. limon* planted in the North-Eastern part of India cleared that limonene (55.4%) and neral (10.39%) were found as primary components, followed by *trans*-verbenol (6.43%) and decanal (3.25%) (Paw et al., 2020). Furthermore, *cis*-α-bergamotene (1.6%), geraniol (1.48%), *trans*-carveol (1.33%), nonanal (1.19%), linalool (1.16%), and α-terpineol (1.07%) were noticed in the peel’s main EOs (Paw et al., 2020). Limonene was the major constituent of the EOs from *C. limon* peels (58.58–63.15%), followed by γ-terpinene (11.19–12.71%), β-pinene (8.56–9.01%), β-myrcene (2.66–3.43%), and α-pinene (2.64–3.11%) (Michaelakis et al., 2009; Golmakani and Moayyedi, 2015; Ahmad et al., 2022). Limonene (59.1%), γ-terpinene (9.7%), β-pinene (5.2%) and β-bisabolene (3.6%). They were the main specific composition from fresh and dried peels of *Citrus* × *limon* cv. Femminello Comune (Rutaceae) from Rocca Imperiale (Italy) (Loizzo et al., 2016). Additionally, the greatest components in the most important leaf oils were limonene (27.58%), β-pinene (17.1%), geranial (7.4%), neral (6.67%) and sabinene (5.1%) (Loizzo et al., 2016). Leaf primary EO and peel from the Cretan variety Zambetakis (*C. limon*) demonstrated the existence of limonene as a principal component, and β-pinene, myrcene, neral, geranial, neryl acetate, geranyl acetate, and β-caryophyllene have been identified in the leaf EO, while the peel EO contained γ-terpinene, β-pinene, myrcene, neral, and geranial (Vekiari et al., 2002).

During the current study, the effect of *C. limon* EOs from leaves and peels that were treated three times with AgNPs during the harvest season were tested against the rice weevil, *S. oryzae*, which caused significant damage to several stored grains, as reported previously (Abdelsalam et al., 2019c; El-Naggar et al., 2020; Yang et al., 2020; Mosa et al., 2021; Alsalem et al., 2022), the infection by this type of insects caused reductions in the nutritional quality and grain yield weight, in addition, their effect on human health. The toxic effects of EOs such as lemon involve several factors, including, inhaled, ingested, or absorbed and which may have contact, fumigation, and the variations in toxicity were found to depend upon the kind of the assessed EOs. The essential oils, particularly those of spearmint and clove, can be used as efficient control agents for stored grain insects by fumigation. Also, we utilized these natural materials against *S. oryzae* to minimize the usage of the chemical insecticides that happened hazard influence humans and the environment (Fields and White, 2002; Ducom, 2012; Maletsema Alina, 2016; Pavlidi et al., 2018; Chelef et al., 2020; Abualnaja et al., 2021b). The achieved results clarified that the efficiency of the lemon EOs was directly related to concentration and response. Using effective and eco-friendly materials such as EOs and plant extracts is an alternative method to control the stored product insects in many crops after harvest (Koul and Walia, 2009; Mamun and Ahmed, 2011; Mackled et al., 2019; Mahomed et al., 2019; Mosa et al., 2021). The current results showed insecticidal activity for the lemon leaves and peel EOs against the rice weevils, and its recommended use and these results are in harmony with previous studies (El-Akhal et al., 2014; Abdelgaleil et al., 2015). As recommended, *C. limon* EOs from leaves and peels could be used as insecticides for the rice weevils (Ali et al., 2017; Gucwa et al., 2018; Abbas et al., 2022; Abdelsalam et al., 2022).

Several studies on the antifungal activity of citrus EOs have been published in the literature (Sharma and Tripathi, 2008; Viuda-Martos et al., 2008). EOs from lemon leaves or peels were found to have the ability to decrease or inhibit the growth of *F. solani* fungus *in vitro* at appropriate concentrations. *F. solani* radial development was substantially reduced to varying degrees; EOs of lemon treatments with mild inhibiting minimal doses had a more pronounced action. Monoterpenes are thought to be responsible for their high biological activity. Citrus EOs were shown to have a significant concentration of monoterpenes, including limonene, linalool, α-pinene, and terpinene. Several researchers attributed the antifungal activity of citrus EOs to the existence of monoterpenes like linalool, limonene, or central, among others (Sharma and Tripathi, 2006, 2008; Viuda-Martos et al., 2008). They act on hyphae mycelia, causing the cellular wall of the hyphae to lose stiffness and integrity, culminating in the killing of mycelium (Sharma and Tripathi, 2008; Morsy et al., 2022). Several scientists have described the ability of citrus EOs to suppress the development and development of spores (Sharma and Tripathi, 2006, 2008; Chutia et al., 2009; Grbiæ et al., 2011; Abualnaja et al., 2021a; Elkobrosy et al., 2022). The argument that the lemon EOs increased the formation of *Fusarium* spp. spores was notable. It was remarked that correlating the fungitoxic activity of simple compounds or classes of chemicals is difficult (French, 1985). In EOs, many components can work together, and the combination of several compounds can promote the development of fungus spores. The findings support further investigation, highlighting the antifungal capabilities of the principal components of EOs on the one hand, and their ability to inhibit the fungi examined *in vivo* on the other.

## Conclusion

The foliar spraying of AgNPs significantly increased the vegetative growth parameters, flower, and fruit set percentage, and yield in kg per tree and hectare compared to control in the two seasons. The effect of AgNPs was gradually increased in parallel to the increase in the used concentration, reaching 10 mg/L, which was higher than 7.5, which was frequently higher than 5 mg/L. The AgNPs treatment effect was higher in the second season than in the first one. GC–MS analysis of leaves and peel EOs showed the presence of several compounds such as neral, geranial, neryl acetate, D-limonene, geraniol acetate, linalool, and citronellal. The results showed a high effect of lemon peels rather than leaves against *Sitophilus oryzae*. On the contrary, lemon leaf EOs’ antifungal activity was more potent than peel EOs against *Fusarium solani* (acc # OL410542).

## Data Availability Statement

The original contributions presented in the study are included in the article/supplementary material, further inquiries can be directed to the corresponding author/s.

## Author Contributions

WM, MM, NA, SB, and MS: conceptualization, formal analysis, resources, visualization, data curation, and writing—original draft preparation. WM, MM, NA, SB, AA-A, and MS: methodology and validation. NA, SB, and AA-A: software. WM and NA: investigation. WM, MM, NA, SB, MS, AA-A, AB, AA, and ME: writing—reviewing and editing. MS: supervision. WM: project administration. AA-A, AB, AA, and ME: funding. All authors contributed to the article and approved the submitted version.

## Conflict of Interest

The authors declare that the research was conducted in the absence of any commercial or financial relationships that could be construed as a potential conflict of interest.

## Publisher’s Note

All claims expressed in this article are solely those of the authors and do not necessarily represent those of their affiliated organizations, or those of the publisher, the editors and the reviewers. Any product that may be evaluated in this article, or claim that may be made by its manufacturer, is not guaranteed or endorsed by the publisher.
